# Association of gut microbiota composition and function with a senescence-accelerated mouse model of Alzheimer’s Disease using 16S rRNA gene and metagenomic sequencing analysis

**DOI:** 10.18632/aging.101693

**Published:** 2018-12-18

**Authors:** Weijun Peng, Pengji Yi, Jingjing Yang, Panpan Xu, Yang Wang, Zheyu Zhang, Siqi Huang, Zhe Wang, Chunhu Zhang

**Affiliations:** 1 Department of Integrated Traditional Chinese & Western Medicine, The Second Xiangya Hospital, Central South University, Changsha, Hunan 410011, China; 2 Department of Integrated Traditional Chinese & Western Medicine, Xiangya Hospital, Central South University, Changsha, Hunan 410008, China; 3 Department of Gastroenterology, Xiangya Hospital, Central South University, Changsha, Hunan 410008, China

**Keywords:** Alzheimer’s disease, gut microbiome, metagenomics, 16S rRNA sequencing, metabolism

## Abstract

Although an intriguing potential association of the gut microbiome with Alzheimer's disease (AD) has attracted recent interest, few studies have directly assessed this relationship or underlying mechanism. Here, we compared the gut microbiota composition and functional differentiation of senescence-accelerated mouse prone 8 (SAMP8) mice with control senescence-accelerated mouse resistant 1 (SAMR1) mice using 16S rRNA gene and metagenomic sequencing analysis, respectively. Specifically, 16S sequencing results showed that the SAMP8 mice displayed a characteristic composition of the gut microbiome that clearly differed from that of the SAMR1 mice. Moreover, network analysis revealed that the gut microbiota of SAMP8 mice had decreased correlation density and clustering of operational taxonomic units. Metagenomic results revealed that the predominant Cluster of Orthologous Groups functional category related to these changes was the metabolism cluster in SAMP8 mice. The Kyoto Encyclopedia of Genes and Genomes (KEGG) annotation further demonstrated enrichment of the relative abundance of some dominant metabolism-related KEGG pathways in the SAMP8 mice, consistent with the suggested pathogenic mechanisms of AD. In conclusion, this study suggests that perturbations of the gut microbiota composition and the functional metagenome may be associated with AD. Further studies are warranted to elucidate the potential new mechanism contributing to AD progression.

## Introduction

Alzheimer’s disease (AD) is a neurodegenerative disease, characterized by the progressive development of cognitive impairment, representing one of the greatest health challenges worldwide [1]. Despite the fact that substantial progress has been made over the last decade, the AD-related molecular and cellular pathogenesis remain poorly understood [2], and no pharmacologic therapies are available to stop the disease progression [3]. Moreover, the currently available treatments have extremely limited therapeutic effect [4]. Therefore, further investigation into the pathophysiology and molecular mechanisms underlying AD is urgently required.

In recent decades, the potential role of the gut microbiome in altering the health status of the host has attracted considerable attention. An increasing number of studies suggest that gut microbiota, notably the intestinal microbiota, is associated with several diseases such as type 1 diabetes mellitus [5,6], Behcet’s disease [7], hypertension [8,9], schizophrenia [10], and Parkinson’s disease [11]. Moreover, many converging lines of evidence suggest that gut microbiota dysbiosis plays a major role in the development of AD-related pathogenesis [12]. For example, alteration of the gut microbiome was observed in AD transgenic mice [13–17], AD patients [18,19], and transgenic AD *Drosophila* [20]. However, all of these previous studies were based on a 16S rRNA gene sequencing method to determine the microbiota composition, which has known limitations such as the potential for skewing the results owing to amplification bias [21] and inability to identify most microbes at the species and strain level [22]. Alternatively, the development of metagenome sequencing technology can provide a higher resolution of the taxonomic profile with functional classification of the microbiome than possible with 16S rRNA sequencing [23]. However, no study has yet conducted metagenome sequencing analysis of the gut microbiome in relation to AD.

Accordingly, in the present study, to further understand the role of the gut microbiome in AD, we compared the composition and profile of the gut microbiome from fecal samples between senescence accelerated mouse prone 8 (SAMP8) mice, a well-established deterministic model of AD, and control senescence-accelerated mouse resistant 1 (SAMR1) mice using both 16S rRNA gene sequencing and metagenomics sequencing analysis.

## RESULTS

### Cognitive performance of SAMP8 and SAMR1 mice

**Figure 1:**
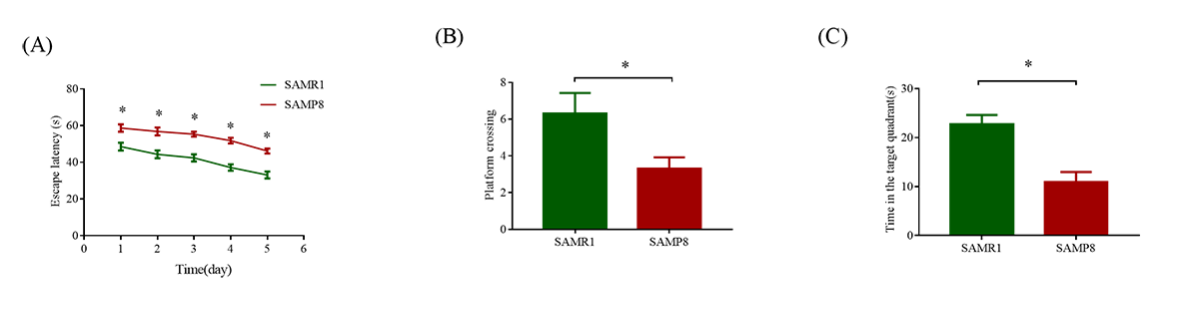
**MWM test used to evaluate the learning and memory ability in SAMP8 and SAMR1 of 8-month-old mice**. (**A**) Mean escape latency in the hidden platform test. (**B**) Number of crossings in the probe trial test. (**C**) Time spent in the target quadrant in the probe trial test. The data were presented as the mean ± SEM; *P < 0.05.

As shown in Fig. 1A, compared with the SAMR1 mice, the mean escape latency of SAMP8 mice was significantly increased (*P* < 0.05). In the probe trial, the SAMP8 mice randomly swam in the tank without knowing the target location, whereas the SAMR1 mice preferentially searched for the target quadrant (Fig. 1B). Moreover, the number of crossings and the time spent in the target quadrant significantly decreased in the SAMP8 mice compared with those in the SAMR1 mice (*P* < 0.05; Fig. 1C). These results confirmed that the SAMP8 mice have severe cognitive impairments even at 8 months of age.

### Gut microbiome composition of SAMR1 and SAMP8 mice using 16S rRNA sequencing

After size filtering, quality control, and chimera removal, a total of 959,692 high-quality sequences were obtained from fecal samples of 26 mice (13 SAMR1 and 13 SAMP8). In addition, 560 OTUs were matched, including 10 phyla, 206 species, and 103 genera of gut microbes that were annotated for subsequent analyses.

**Figure 2:**
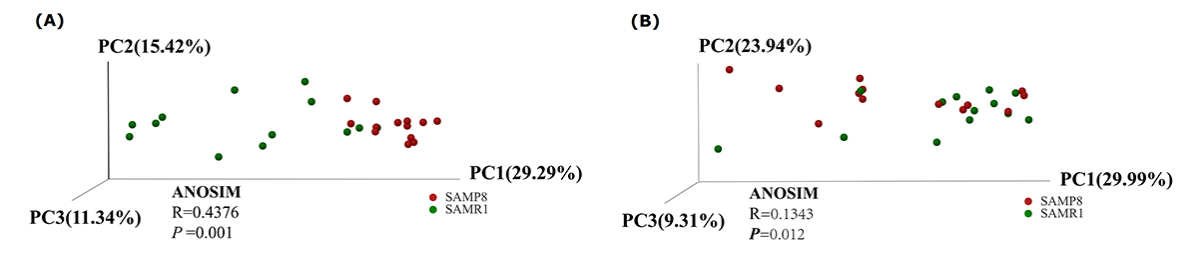
**Principal Coordinates Analysis of (A) unweighted and (B) weighted UniFrac distances for SAMR1 and SAMP8.** The red circles represent the SAMP8 mice (*n* = 13) and green circles represent SAMR1 mice (*n* = 13). PC1, PC2 and PC3 represent the top three principal coordinates that captured the maximum diversity.

To evaluate alterations in the microbiota community structure between SAMP8 and SAMR1 mice, we measured the microbial alpha diversity, using the Chao, Shannon, and ace diversity indices, which showed no significant differences between the groups (*P* = 0.505, 0.9183, and 0.5727, respectively, data not shown). By contrast, the beta diversity analysis showed that the total diversity captured by the top three principal coordinates was 56.05% and 63.24% for unweighted and weighted UniFrac, respectively, and that the microbiota composition of SAMP8 mice was significantly different from that of SAMR1 mice. (ANOSIM R = 0.4376, *P* = 0.001; and R = 0.1343, P = 0.012, for unweighted and weighted distances, respectively, Fig. 2A and B).

**Figure 3:**
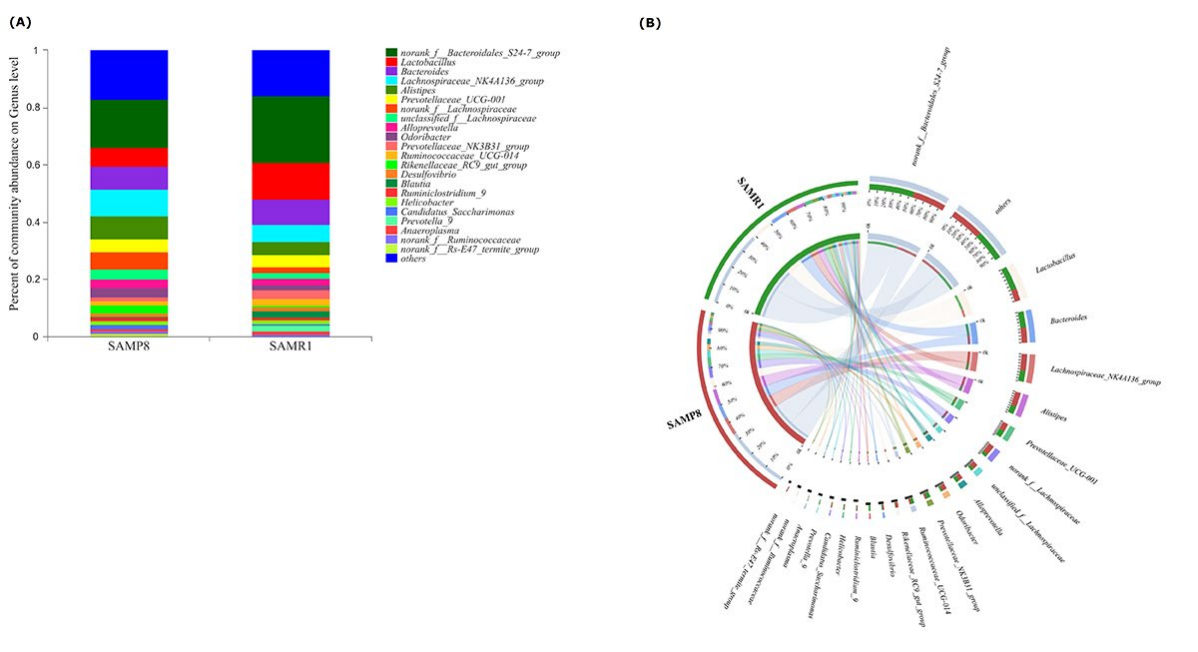
**Relative abundance of microbial community for each group at genus level.** (**A**) Bar-plot analysis shows the average relative abundance of fecal microbiota in each group. (**B**) Circos analysis displays the corresponding abundance relationship between samples and bacterial communities.

To illustrate the differences in the microbiota composition between SAMP8 and SAMR1 mice, we conducted bar plot, Circos, and pie-plot analyses. The bar plot roughly indicated that the relative abundance of different genera varied among the 26 fecal samples at the genus levels. As shown in Fig. 3A, five genera were predominant in fecal samples from both SAMP8 and SAMR1 mice, including *norank_f__Bacteroidales_S24-7_group* (17.13% vs 23.10%), *Lactobacillus* (6.07% vs 12.91%), *Bacteroides* (8.24% vs 9.45%), *Lachnospiraceae_NK4 A136_group*(8.83% vs 5.75%), and *Alistipes* (7.73% vs 4.59%). Circos analysis was used to visualize the corresponding abundance relationship between samples and bacterial communities at the genus level, which confirmed the bar plot analysis results (Fig. 3B).

**Figure 4:**
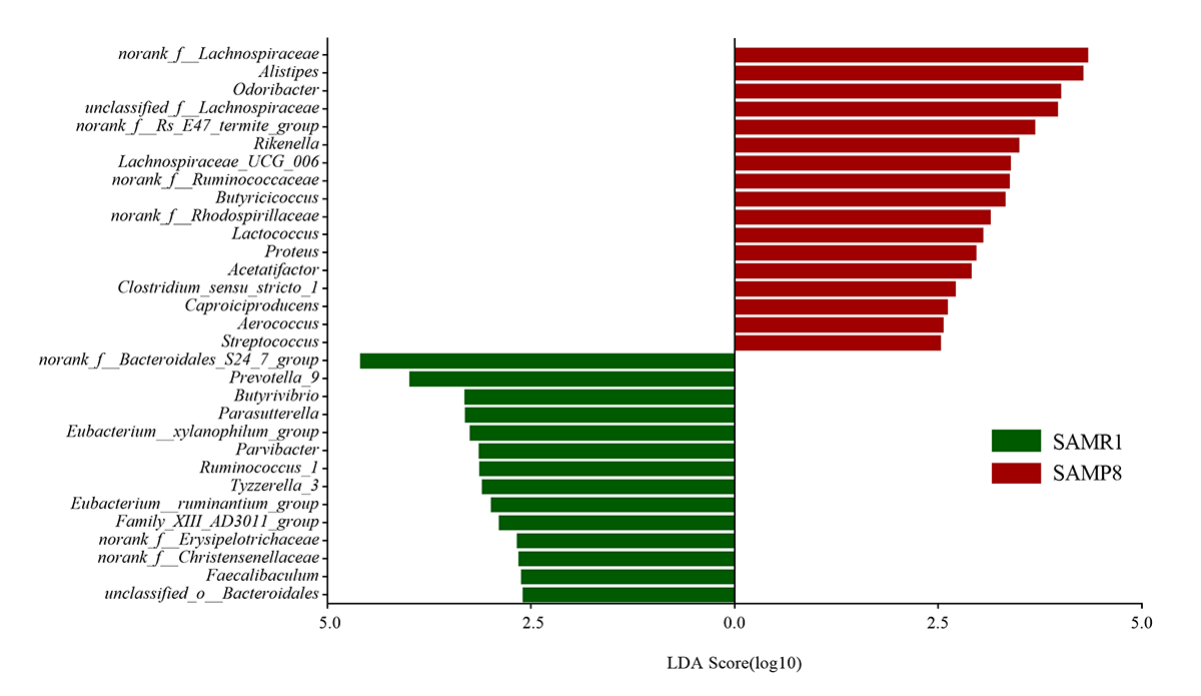
**Gut microbiota bacterial comparisons between SMAP8 and SAMR1 groups analyzed by LEfSe (LDA> 2.5, P< 0.05).** Histogram of the LDA scores for differentially abundant genera. LDA scores were calculated by LDA effect size, using the linear discriminant analysis to assess effect size of each differentially abundant bacterial taxa.

To further determine whether specific individual bacterial taxa were differentially enriched in SAMP8 mice compared with SAMR1 mice, we applied the LEfSe analysis method, which uses LDA coupled with effect size measurements. As shown in Fig. 4, this analysis identified 34 genera, which were differentially abundant between the SAMP8 and SAMR1 mice. For example, a significant enrichment in *norank_f__Lachnospiraceae*, *Alistipes, unclassified_f__Lachnospiraceae* and *Akkermansia,* and *Odoribacter* was identified in SAMP8 mice. While, *norank_f__Bacteroidales_S24_7_group, Prevotella_9, Parasutterella,* or *Butyrivibrio* were significantly more abundant in fecal samples from SAMR1 mice.

**Figure 5:**
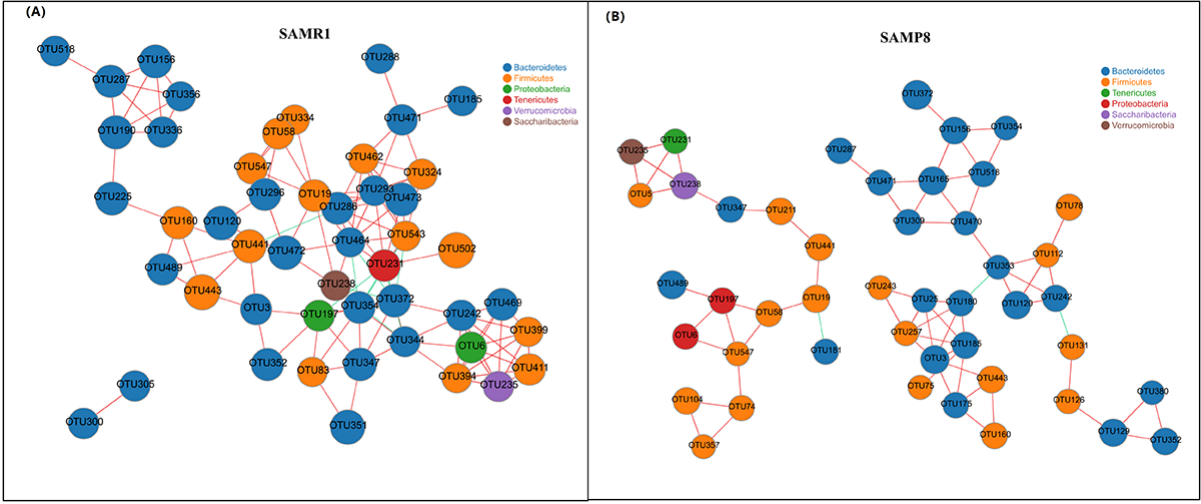
**Correlation network analysis of the 50 most abundant OTUs for (A) SAMR1 and (B) SAMP8**. Networks show significant positive (red) and negative (green) pairwise correlations between operational taxonomic units (OTUs). OTUs are colored by phylum affiliation and sized by mean relative abundance.

Finally, we performed a correlation network analysis to evaluate if SAMP8 was associated with changes in the correlation structure and putative interaction structure of the gut microbiota and then to identify the putative keystone genera. We found that networks constructed from samples of SAMP8 mice had fewer edges(138 vs. 242), a lower mean degree (3 vs. 5), and a lower transitivity (0.516 vs. 0.579), indicating that there were fewer significant correlations and less clustering of OTUs compared to samples from SAMR1 mice (Fig. 5A and B). Moreover, degree (DC), closeness (CC), and betweenness (BC) centrality were computed to evaluate the taxa importance within the network. Based on the high scores of these topological properties (arbitrarily determined as DC>0.1, CC>0.2 and BC>0.1), 4 OTUs were selected (OTU353, OTU180 for SAMP8, and OTU441, OTU286 for SAMR1), representing putative keystone genera within this network.

### Metagenomic analysis revealed different functional profiles between SAMR1 and SAMP8 mice

To investigate the functional profile of the gut microbiome in SAMR1 and SAMP8 mice, we also performed metagenomic analysis of the microbial DNA extracted from fecal samples (four mice per group). A total of 602,400,188 filtered reads (89.5 Gb) and 6,220,223 ORFs were used for functional annotation in the COG and KEGG databases.

**Figure 6:**
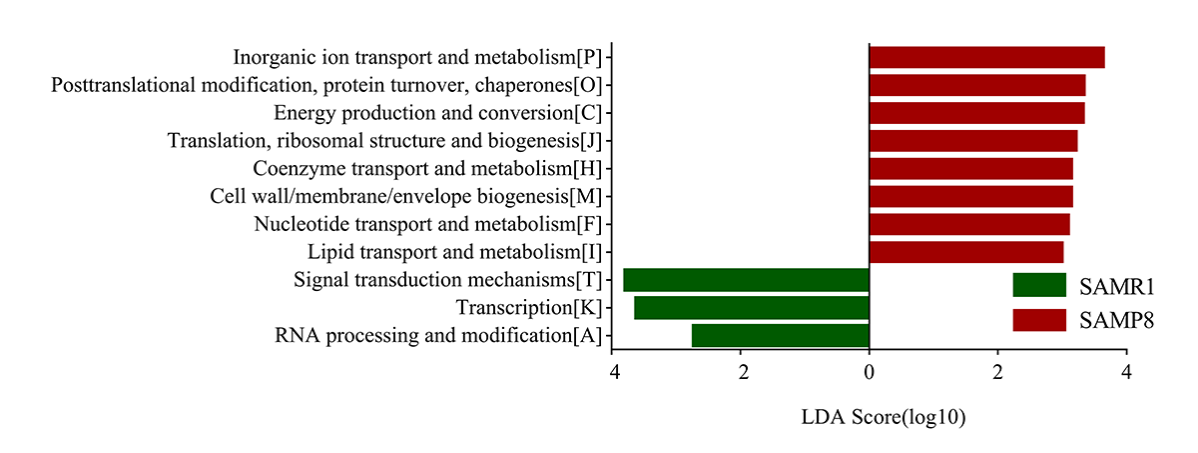
**COG category differences in metagenome between the SAMP8 and SAMR1 analyzed by LEfSe analysis (LDA> 2.5, P< 0.05).** Histogram of the LDA scores for differentially abundant COG categories.

To determine biologically significant differences, LEfSe analysis was also conducted to detect the functional COG categories with significantly diﬀerent abundances between SAMR1 and SAMP8 mice. As shown in Fig. 6, 11 functional COG categories were observed with significantly overabundant reads in the SAMP8 group, which were assigned to the lipid transport and metabolism [I]; nucleotide transport and metabolism [F]; cell wall/membrane/envelope biogenesis [M]; coenzyme transport and metabolism [H]; translation, ribosomal structure and biogenesis [J]; energy production and conversion [C]; posttranslational modification, protein turnover, chaperones [O]; and inorganic ion transport and metabolism [P] categories. By contrast, the SAMR1 group had more reads involved in the RNA processing and modification [A], transcription [K], and signal transduction mechanisms [T] categories. Overall, the results could be summarized into three categories: information storage and processing (cluster I), cellular processes and signaling (cluster II), and metabolism (cluster III). Notably, the metabolism cluster (cluster III) was predominant in SAMP8 mice, and was related to inorganic ion transport and metabolism; lipid transport and metabolism; coenzyme transport and metabolism; energy production and conversion; and nucleotide transport and metabolism.

**Figure 7:**
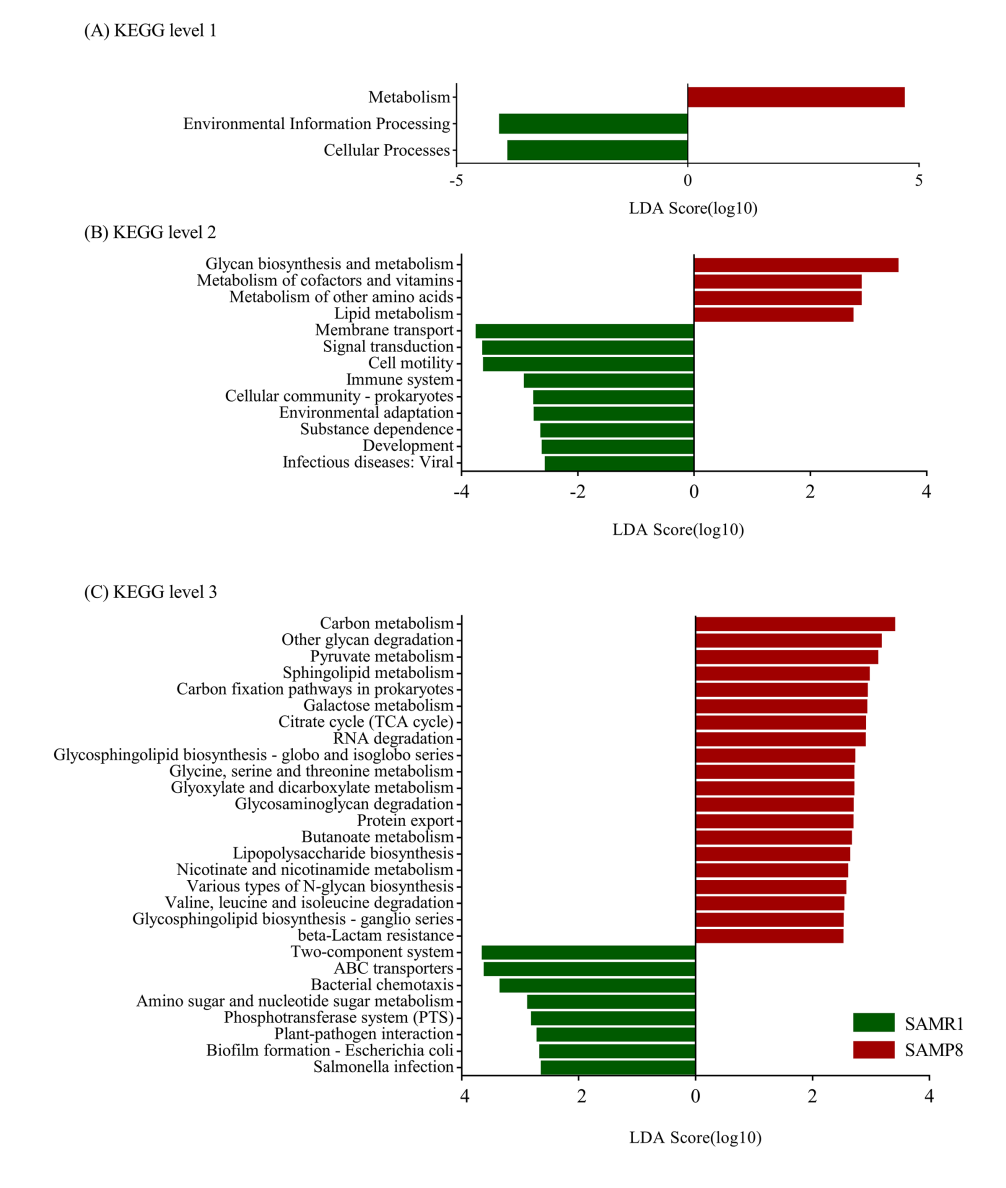
**Plots of KEGG pathways comparisons between SAMP8 (green) and SAMR1 (red) at levels 1 (A), 2 (B), and 3 (C) analyzed by LEfSe analysis (LDA> 2.5, P< 0.05**). Histogram of the LDA scores for differentially abundant KEGG pathway.

Furthermore, we determined changes in functional composition using the KEGG pathway database. LEfSe analysis was then also performed to explore KEGG pathways with significantly diﬀerent abundances between SAMR1 and SAMP8 mice. Using the threshold values (LDA> 2.5, P< 0.05), we found that at KEGG level 1 (Fig. 7A), the proportion of sequences associated with metabolism was significantly increased in SAMP8 mice, while environmental information processing and cellular processes significantly declined. At level 2 (Fig. 7B), the functional categories related to glycan biosynthesis and metabolism, metabolism of cofactors and vitamins, metabolism of other amino acids, and lipid metabolism were enriched in the fecal microbiome of SAMP8 mice. At level 3 (Fig. 7C), we found that 20 KEGG pathways (including carbon metabolism, other glycan degradation, pyruvate metabolism, sphingolipid metabolism, carbon fixation pathways in prokaryotes, *et al.*) were significantly enriched in SAMR8 mice, and six KEGG pathways (including two-component system, ABC transporters, bacterial chemotaxis, amino sugar and nucleotide sugar metabolism, Phosphotransferase system, *et al.*) were significantly increased in SAMR1 mice (LDA> 2.5, P< 0.05). Interesting, in the SAMR8 mice, multiple functional pathways that were more highly represented were also involved in metabolism.

**Figure 8:**
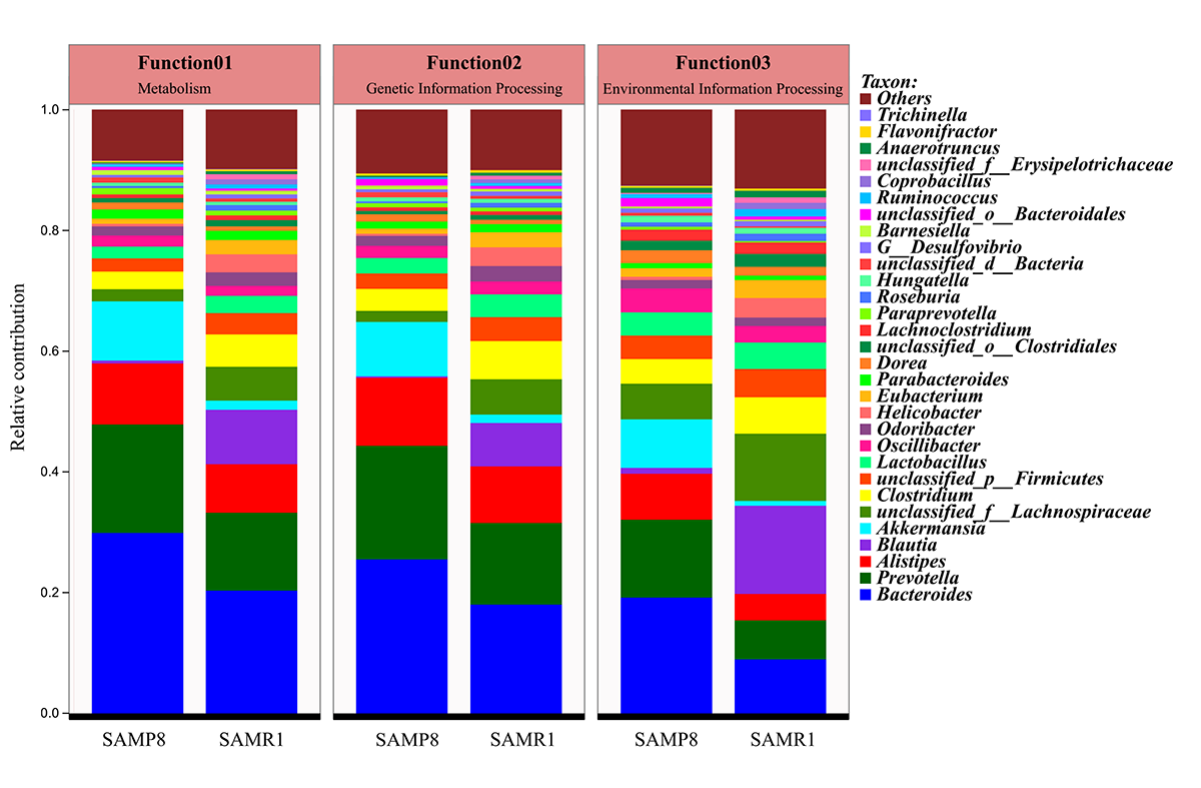
Comparison of functional genes related to KEGG pathways at level 1 and their contributing species in SAMP8 and SAMR1.

In addition, the top 10 genera, including *Oscillibacter*, *Lactobacillus*, *unclassified_p__Firmicutes*, *Clostridium*, *unclassified_f__Lachnospiraceae*, *Blautia*, *Akkermansia*, *Alistipes*, *Prevotella*, and *Bacteroides*, mainly contributed to differences in KEGG level-1 pathways between SAMP8 and SAMR1 mice (Fig. 8). Among them, the relative abundance of *Prevotella* and *Bacteroides* was increased, while the abundances of *unclassified_f__Lachnospiraceae*, *Clostridium*, and *Blautia* decreased in SAMP8 compared to SAMR1 mice.

## DISCUSSION

To our knowledge, this is the first examination of specific patterns of gut microbiota composition and function in a transgenic mouse model of AD using both 16S rRNA gene and metagenomics sequencing of fecal samples.

In contrast to a previous study using SAMP8 mice [17], we did not detect a significant difference in microbiota alpha diversity compared to control SAMR1 mice, which might be partially due to differences in age between the mice used in the two studies. Furthermore, a previous report showed no significant difference in gut microbe diversity and richness in female R6/1 transgenic mouse model of Huntington's disease [24] or patients with major depressive disorder [25]. Moreover, increases in alpha diversity in male R6/1 transgenic mouse [24], AD *Drosophila* [20], and APP/PS1 transgenic mouse of AD [26] have been previously detected. Therefore, the role of microbial diversity in AD function remains a subject of debate requiring further investigation [27].

To tackle this question, we first measured microbial beta-diversity to determine the similarity in the overall community structure between samples [28], demonstrating a significant difference in microbiota community structure between SAMP8 and SAMR1 mice, which was confirmed by the 16S rRNA sequencing data. SAMP8 microbial dysbiosis was mainly characterized by altered abundances of five genera, with a significant decrease in the relative abundance of the predominant genus *norank_f__Bacteroidales_S24-7_group*. This genus belongs to the family *Bacteroidales_S24–7_group*, which was also shown to be depleted in mice fed a high-fat diet [29], and plays a role in electron transport and oxidative stress to mediate host-microbe interactions [30]. We also found higher *norank_f__Lachnospiraceae* and *unclassified_f__Lachnospiraceae* abundance in SAMP8 mice, which is consistent with a previous study showing that the abundance of *Lachnospiraceae* was increased in patients with AD or mild cognitive impairment [18,31]. However, another study found that the relative abundance of *Lachnospiraceae* was significantly decreased in patients with AD [19]. These inconsistent results warrant further validation and investigation. By contrast, the abundance of the genera *Alistipes* (family *Rikenellaceae*) and *Odoribacter* (family *Odoribacteraceae*) significantly increased in SAMP8 mice, which have also been found to be more abundant in AD patients [18] and in APP/PS1 transgenic mouse model of AD [15]. In addition, the relative proportion of *Rikenellaceae* was reported to be significantly higher in patients with major depressive disorder [27].

To determine whether the structure of the gut microbiota is also altered in SMAP8, we performed a correlation network analysis and found that there were fewer correlations, smaller betweenness centrality and less clustering of OTUs in SAMP8 than in SAMR1. These suggested that the altered network structure in SAMR8 may be involved in the decreased fermentation capacity of the gut microbiota.

Metagenomic sequencing was then used to determine the functional features of the microbiota between the two groups. The COG annotations in SAMP8 mice were mainly involved in metabolism, including inorganic ion, coenzyme, nucleotide, and lipid transport, and metabolism. This observation is compatible with the hypothesis that AD is fundamentally a metabolic disease, and patients with AD often display a coexisting metabolic disorder phenotype in conjunction with the neurodegenerative pathology [32,33]. The KEGG pathway analysis [34] further indicated that these perturbed gut bacteria in SAMP8 mice were strongly associated with dysregulation of basic metabolic processes such as lipid metabolism, carbon metabolism, and pyruvate metabolism. Thus, both the COG and KEGG analyses indicated that alternations of gut microbiota might contribute to AD pathogenesis through metabolic pathways. These results are consistent with those of previous studies [14,18]. For example, lipid metabolism in the central nervous system has been suggested to be an important factor contributing to the pathogenesis of AD, considering the identification of the apolipoprotein E gene as a genetic risk factor for the disease [35]. Prevailing data suggest that abnormal lipid metabolism influences amyloid-beta (Aβ) metabolism and deposition in both the brain parenchyma and vasculature, as well as tau hyperphosphorylation and aggregation, which is then likely to trigger a series of downstream catalytic events that eventually affect the progression of the pathogenesis of AD [36]. Moreover, aberrant pyruvate metabolism plays an especially prominent role in cancer, heart failure, and neurodegeneration [37]. Pyruvate was also shown to prevent the development of age-dependent cognitive deficits in a mouse model of AD without reducing amyloid and tau pathology [38]. In addition, many lines of evidence have recently emerged to suggest that carbohydrate metabolism is disordered in AD, which contributes to initiation of the dementia. Beside these metabolic pathways, ABC transporters are dysregulated in SAMP8 mice, which constitute one of the largest protein families that are widely distributed and evolutionarily conserved, and are involved in detoxification and transport processes [39].

There are some limitations of the present study that should be mentioned to place the findings in context [40]. First, because of the relatively small number of samples used to generate microbiota and metagenomic data, these results should be confirmed in a validation clinical cohort. Second, we used the fecal microbiome to infer changes of the gut microbiome, and only focused on microbiota composition and function; thus, metabolomics and metatranscriptomics data are needed to explore these preliminary findings in further detail. In addition, the host functions were not assessed, which may largely drive the observed microbiome changes. Moreover, associations between the most relevant taxa and AD were not validated by real-time quantitative PCR. Thus, further studies will be necessary to clarify the effect of these limitations on the present findings.

Nevertheless, this work provides new insight into differences in the composition and function of the gut microbiota between SAMP8 and SAMR1 mice, revealing dynamic alterations in fecal microbiota that correlated with known changes occurring in the AD pathologic processes. Moreover, these perturbed gut bacteria were strongly associated with changes of several gut microflora-related metabolites, indicating that AD progression is associated with disturbance of gut bacteria at the abundance level and also substantial alteration of the multiple metabolic pathways. These findings may provide new mechanistic insights regarding the role of perturbations of the gut microbiome in AD development and progression. The present study also suggests that analysis of the role of the gut microbiome in disease benefits from functional gene analysis compared to simple comparison of the microbial community.

## MATERIALS AND METHODS

### Animals

Male 6-month-old SAMP8 and age-matched SAMR1 mice were purchased from the First Teaching Hospital of Tianjin University of Traditional Chinese Medicine (Tianjing, China). The animals were kept under standard conditions of temperature (24 ± 1°C) and humidity with a 12-h light/dark schedule, with food and water available *ad libitum*. All animal experiments were conducted in compliance with the Guide for the Care and Use of Laboratory Animals and were approved by the Ethics Committee of Central South University (Changsha, China).

### Morris water maze (MWM) test

The spatial learning and memory abilities of SAMP8 and SAMR1 mice were assessed by the MWM test at 8-month-old, as previously described with minor modifications [41]. In brief, to test the spatial learning capacity, the mice were submitted to four trials per day for five consecutive days in a circular pool (120 cm diameter and 50 cm height) containing a 10-cm-diameter hidden platform submerged 1 cm below the water surface. At each trial, the mouse was placed into the water, facing the pool wall, and given 60 s to locate the platform. If the mice failed to locate the platform within 60 s, they were guided to it and allowed to remain for 15 s. On the sixth day, the platform was removed, and then the mice were allowed to swim for 60 s. All trials were monitored by an overhead video camera connected to the ANY-maze video tracking system (Stoelting Co., USA).

### Feces collection

Fresh mouse feces were collected into individual sterile EP tubes, quickly frozen on dry ice, and then transferred into an −80°C cryogenic freezer for cryopreservation until DNA extraction.

### 16S rRNA gene sequencing analysis

The gut microbiota of the mice was first determined with 16S rRNA sequencing analysis as described previously [42,43]. Briefly, the microbial DNA was extracted from 26 fecal samples (13 from SAMP8 mice and 13 from SAMR1) using E.Z.N.A.® Stool DNA Kit (Omega Bio-tek, Norcross, GA, USA) in accordance with the manufacturer’s protocols. Then, PCR amplification of the V3-V4 hypervariable regions of the bacterial 16S rRNA gene was performed using universal primers (338F 5′-ACTCCTACGGGAGGCAGCAG-3′, 806R 5′-GGACTACHVGGGTWTCTAAT-3′) incorporating the FLX Titanium adaptors and a barcode sequence. Subsequently, purified amplicons were pooled in equimolar amounts, and paired-end sequenced on an Illumina MiSeq platform (Illumina, San Diego, USA) according to standard protocols described by Majorbio Bio-Pharm Technology Co. Ltd. (Shanghai, China). Raw FastQ files were demultiplexed, quality-filtered by Trimmomatic, and merged using FLASH. Trimmed sequences were clustered to operational taxonomic units (OTUs) with a 97% similarity cut-off using UPARSE (version 7.1 http://drive5.com/uparse/), and chimeric sequences were identified and removed using UCHIME. The taxonomical assignment of OTUs was performed by the RDP Classifier algorithm (http://rdp.cme.msu.edu/) against the Silva database (https://www.arb-silva.de/) using a confidence threshold of 70%.

### Metagenomic analysis

The mouse gut microbiota was further investigated with a metagenomic sequencing method as described previously [44]. Total genomic DNA was extracted from 8 fecal samples (4 from SAMP8 mice and 4 from SAMR1) using the E.Z.N.A® Stool DNA kit (Omega Bio-Tek, USA) following the manufacturer’s instructions. DNA was fragmented to an average size of approximately 300 bp using TruSeq™ DNA Sample Prep Kit with Covaris M220 (Gene Company Limited, China) for paired-end library construction. Then, the metagenomic sequencing was performed on an Illumina HiSeq4000 sequencing platform (Illumina Inc., San Diego, CA, USA) at Majorbio Bio-Pharm Technology Co., Ltd. (Shanghai, China) according to the manufacturer’s protocols. The raw sequence reads were trimmed with a quality score lower than 20 and a length shorter than 50 bp. The clean raw reads were then assembled by using the SOAPdenovo software to obtain contigs for the following prediction and annotation. Subsequently, the open reading frames (ORFs) from each sample were predicted using MetaGene (http://metagene.nig.ac.jp/). The cluster of orthologous groups of proteins (COG) annotation of the ORFs was obtained using the eggNOG database (Version 4.5) via BLASTP(BLAST Version 2.2.28+) with an e-value cutoff of 1e^-5^. The Kyoto Encyclopedia of Genes and Genomes (KEGG) pathway annotation was performed using a BLAST search (Version 2.2.28+) against the KEGG database (http://www.genome.jp) at an optimized e-value cutoff of 1e^-5^.

### Bioinformatics analysis

For 16S rRNA gene sequencing analysis, diversity was calculated using the QIIME tool [45]. Differences in alpha diversity were calculated by the Chao, Shannon, and ace diversity indices. Beta diversity was determined using both unweighted and weighted UniFrac phylogenetic distance matrices, and visualized in principal coordinates analysis (PCoA) plots. The statistical significance was evaluated with analysis of similarities (ANOSIM). A collinearity diagram was constructed with Circos software (http://circos.ca/software/download/circos/) to visualize the corresponding abundance relationship between samples and bacterial communities at the genus levels. Statistically significant differences in the relative abundance of genera between mouse strains were performed using linear discriminant analysis (LDA) effect size (LEfSe). Only LDA values > 2.5 at a P value <0.05 were considered significantly enriched. NetworkX was used to explore and visualize the associations between the microbial communities. To describe the topology of the resulting networks, degree (DC), closeness (CC) and betweenness centrality (BC) were calculated [46].

For metagenomic analysis, significantly different in COG and KEGG categories between mouse strains were determined using linear discriminant analysis (LDA) effect size (LEfSe). Only LDA values > 2.5 at a P value <0.05 were considered significantly enriched.

### Statistical analysis

For the MWM tests, data are presented as the mean ± standard error of the mean. One-way or two-way ANOVA analyze was used to evaluate the difference between the groups. *P*< 0.05 was considered statistically significant. All statistical analyses were performed using the SPSS 21.0.
